# Turn to me: electrophysiological correlates of frontal vs. averted view face and body processing are associated with trait empathy

**DOI:** 10.3389/fnint.2012.00106

**Published:** 2012-11-30

**Authors:** Denise Soria Bauser, Patrizia Thoma, Boris Suchan

**Affiliations:** Department of Neuropsychology, Institute of Cognitive NeuroscienceRuhr University Bochum, Germany

**Keywords:** emotional expression, P100, N170, cognitive empathy, affective empathy

## Abstract

The processing of emotional faces and bodies has been associated with brain regions related to empathic responding in interpersonal contexts. The aim of the present Electroencephalography (EEG) study was to investigate differences in the time course underlying the processing of bodies and faces showing neutral, happy, or angry expressions. The P100 and N170 were analyzed in response to the presentation of bodies and faces. Stimuli were presented either from a perspective facing the observer directly or being averted by 45° to manipulate the degree to which the participants had the impression of being involved in a dyadic interpersonal interaction. Participants were instructed to identify the emotional expression (neutral, happy, or angry) by pressing the corresponding button. The result pattern mirrored poorer behavioral performance for averted relative to frontal stimuli. P100 amplitudes were enhanced and latencies shorter for averted relative to frontal bodies, while P100 and N170 components were additionally affected by electrode position and hemisphere for faces. Affective trait empathy correlated with faster recognition of facial emotions and most consistently with higher recognition accuracy and larger N170 amplitudes for angry expressions, while cognitive trait empathy was mostly linked to shorter P100 latencies for averted expressions. The results highlight the contribution of trait empathy to fast and accurate identification of emotional faces and emotional actions conveyed by bodies.

## Introduction

The human body, but especially the face region, provides crucial information about other people's mental states. Successful social interaction depends on the ability to infer other people's emotions, beliefs, and intentions on the basis of their faces and body language. de Gelder et al. (2010) suggest that distinct cortical mechanisms are responsible for the successful extraction of emotional signals from faces and bodies. While facial emotions might be more strongly related to an individual's mental state, perceived bodily emotions rapidly, and automatically provide information about a specific action which might be linked to these emotions. Recent investigations focused on the functional neuroanatomy and the time course associated with the processing of neutral and emotional faces and bodies. However, until now it remains unclear whether this ability and its neuronal correlates are associated with trait empathy.

Functional resonance imaging (fMRI) studies revealed that the fusiform gyrus, the temporoparietal junction, and the amygdala play an important role in the perception of body and face expressions (Morris et al., 1996; Rotshtein et al., 2001; Hadjikhani and de Gelder, 2003; de Gelder, 2006; Grezes et al., 2007; van de Riet et al., 2009; Kret et al., 2011). Kret et al. (2011) directly compared the perception of dynamic facial and body emotions and reported that the extrastriate body area, the motor and premotor cortices, and the superior temporal sulcus were more strongly activated in response to emotional bodies than to faces. Thus, the same areas seem to be activated by emotion perception and emotional action (for a review, see de Gelder et al., 2010). The data also support the assumption that we base our understanding of other people's emotional expressions on the awareness of our own body states. Furthermore, seeing emotional bodies might trigger the activation of areas which are responsible for action preparation.

Perceiving emotions expressed by bodies appears to rely on a network which can be divided into three interconnected emotional circuits (de Gelder, 2006): a subcortical network consisting of the superior colliculus, the amygdala, the pulvinar, and the striatum, a cortical network encompassing the lateral inferior occipital cortex, the superior temporal sulcus, the intraparietal lobule, the fusiform gyrus, the amygdala and the premotor cortex, and an interface system based on the insula, the somatosensory cortex, the anterior cingulate cortex, and the ventromedial prefrontal cortex. The subcortical network is responsible for automatic and reflex-like emotional reactions. The cortical network processes emotions expressed by the body in detail and computes the adequate behavioral response. The interface system might be seen as a body awareness system, which uses the emotional reaction of one's own body to process emotions expressed by other bodies.

In electrophysiological studies, neutral faces and bodies have been shown to elicit specific event-related potentials (ERPs) peaking about 100 and 170 ms after stimulus onset (termed P100 and N170) in occipito-temporal areas (for a review, see Minnebusch and Daum, 2009). Both ERP components are enhanced for faces and bodies compared to other stimulus categories. The P100 is associated with the classification of a stimulus as a face/body (Herrmann et al., 2005) and with the processing of low level stimulus features. Processes related to the structural encoding of a stimulus and the generation of a global stimulus configuration are linked to the N170 (Eimer, 2000a,b; Rossion et al., 2000). The P100 seems to be sensitive to facial and bodily emotions, indicating that the extraction of emotional expressions takes place before the recognition of the person's identity occurs (Eimer, 2000a,b; Meeren et al., 2005; Righart and de Gelder, 2007; van Heijnsbergen et al., 2007). Furthermore, these studies support the assumption that emotional faces and bodies might be processed by similar neuronal mechanisms. These mechanisms process emotional signals rapidly and automatically. However, previous studies used predominantly negative emotions or compared the time course of negative and neutral emotions (e.g., Hadjikhani and de Gelder, 2003; Stekelenburg and de Gelder, 2004; Meeren et al., 2005). It is a matter of debate, whether emotional modulation affects the N170 or not (see Vuilleumier and Pourtois, 2007). Batty and Taylor (2003) found differences between positive, negative, and neutral emotions in the time window of the N170 using faces expressing the six basis emotions. Negative emotions elicited delayed N170 amplitudes compared to positive emotions. In addition, fearful faces elicited an enhanced N170 compared to neutral or surprised faces. However, these effects are not specific to a single emotion as comparable effects were reported for distinct emotional expressions. The present investigation aimed to explore similarities as well as differences in the time course of emotional face and body processing by comparing positive (happy), negative (angry), and neutral emotional expressions.

It is well-known that the same cortical system—involving the so-called mirror neuron system—is responsible for both the production and the perception of emotional expressions (Gallese et al., 2004). Identification of other people's emotional states, thus, seems to rely on our own body, and the evaluation of the emotions conveyed by other peoples' facial and body expressions may trigger empathic reactions. Empathy is currently thought of as a multifaceted construct involving at least three components (e.g., Decety and Jackson, 2004): (1) A cognitive component mediating both the decoding of other people's emotional states and the integration of contextual information about the potential reasons for these emotional states in others. Cognitive empathy is thought to considerably overlap or even to be synonymous with affective mentalizing. (2) An affective component facilitating both the affective sharing of other people's emotional states and one's own affective response to the emotional situation another person is in. (3) A mechanism monitoring the distinction between one's own and the other person's feelings during empathizing. The latter is thought to rely on top-down executive control processes mediating downregulation or enhancement of the empathic response depending on contextual factors (Singer and Lamm, 2009). While the inferior frontal gyrus and the inferior parietal lobule, as parts of the human mirror neuron system, as well as the insula and the anterior cingulate cortex, have been associated with affective empathy, the ventromedial prefrontal cortex, the medial temporal lobe, and the temporo-parietal junction have been related to cognitive empathy and self-other distinction (Shamay-Tsoory, 2011). Thus, there is an overlap between the brain regions mediating empathic reactions and those involved in the perception of facial and bodily emotional expressions, as described above.

The specific interpersonal context is likely to determine the intensity and nature of the empathic reaction and also the neural response associated with it (Singer and Lamm, 2009). One such factor modulating the empathic response might be the degree to which a person feels that she actually might be involved in a direct interaction with another individual. This might e.g., be influenced by whether the other person actually faces the observer directly, suggesting a dyadic interaction. In an fMRI study, Schulte-Rüther et al. (2007) manipulated the head direction of emotional synthetic stimulus faces such that they were either directed to face the observer directly or averted by 45°. They hypothesized that empathic reactions should be triggered more easily in the direct gaze condition. The results suggested that direct gaze affects empathic reactions on the behavioral level, enhancing the empathic response. However, this behavioral effect was not paralleled by differential activations beyond early visual processing areas in the striate cortex. The authors argue that the effect might be too small to detect or otherwise could have reflected differences in the visual characteristics of the stimuli. As time resolution is superior for Electroencephalography (EEG) relative to fMRI techniques, an electrophysiological investigation might reveal delayed processing for averted compared to frontal view emotional bodies and faces, and this effect might further be associated with trait empathy.

Taken together, the aims of the present paper were threefold: first, we aimed to investigate the differences underlying the processing of bodies and faces showing neutral, happy, and angry expressions. Second, we aimed to assess for the first time, whether the electrophysiological and behavioral responses to neutral and emotional faces and bodies are attenuated when the stimuli are displayed from an averted view relative to a frontal view perspective. Third, we aimed to relate this effect, both on a behavioral and on an electrophysiological level, to trait empathy.

## Materials and methods

### Participants

Two separate experiments were performed; one involving face and one involving body stimuli. Seventeen healthy and right-handed undergraduate students (11 female, 19–35 years, mean age: 25.3 ± 4.1 years) participated in the experiment involving face stimuli and 16 healthy and right-handed undergraduate students (13 female, 19–35 years, mean age: 25.8 ± 4.6 years) participated in the experiment involving body stimuli. Eleven subjects participated in both the face and the body experiment. All participants studied Psychology at the Ruhr-University Bochum and had normal or corrected to normal vision. The current study was performed in accordance with ethical standards laid down in the Declaration of Helsinki (Varga, 1975) with approval from the local Ethics board, and written informed consent was obtained from all subjects. Participants were reimbursed with 15 EUR for their participation.

### Stimuli

**Figure 1:**
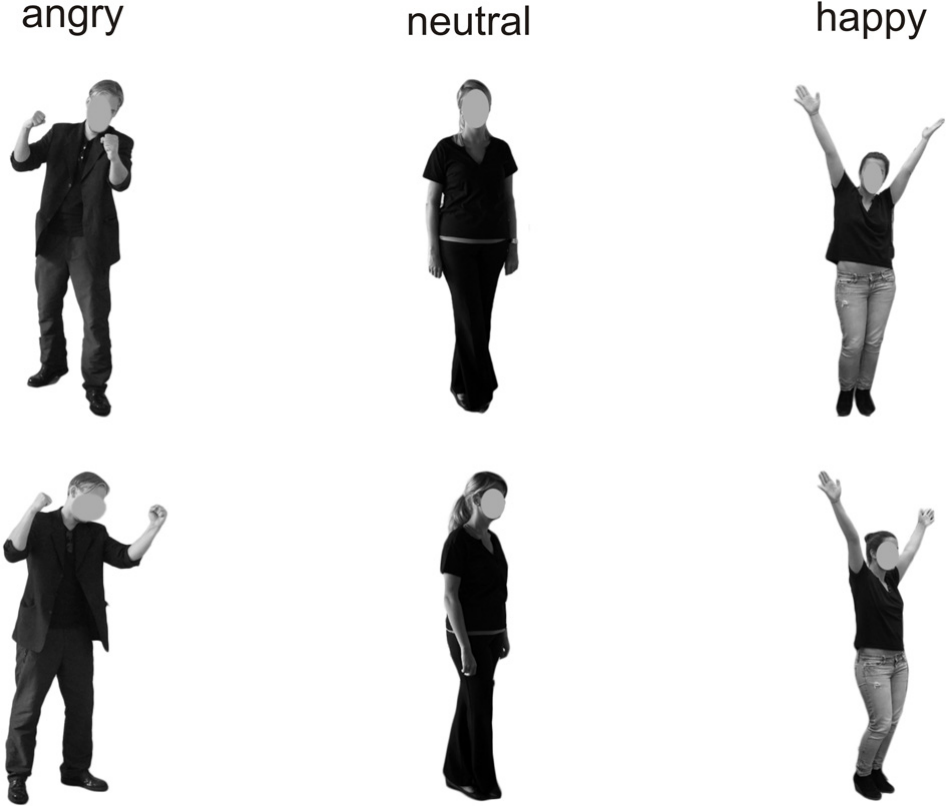
**Examples of the presented stimuli depicting bodies from a frontal (top) and averted (bottom) perspective**.

**Figure 2:**
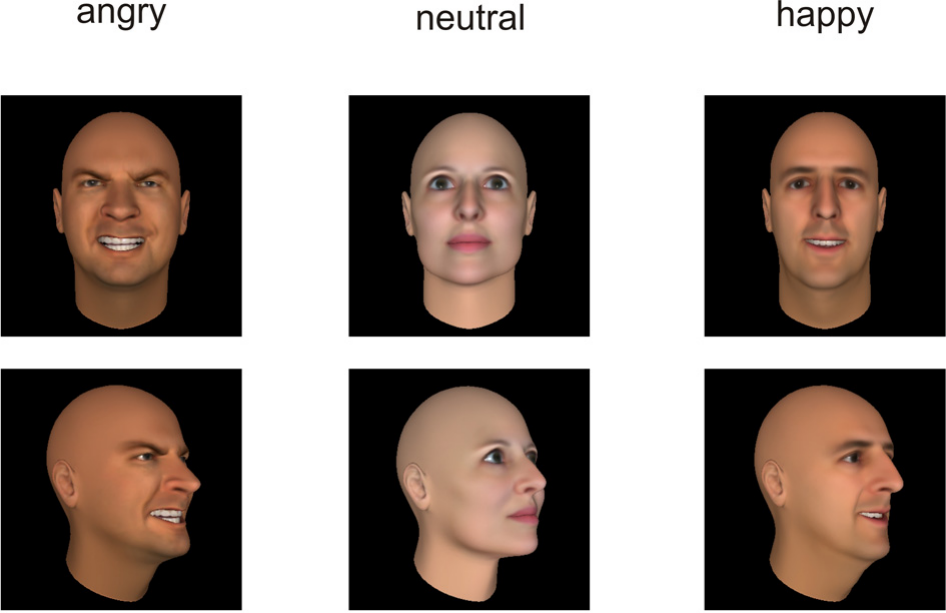
**Examples of the presented stimuli depicting faces from a frontal (top) and averted (bottom) perspective**.

All stimuli were taken from the Bochum Emotional Stimulus Set (BESST: Thoma et al., accepted) database which contains validated photos of real-life bodies with blurred faces as well as pictures of synthetic faces, created using FaceGen Modeller 3.5 software (Singular Inversions, Vancouver, BC, Canada: www.facegen.com). Using virtual facial avatars allows for a greater degree of stimulus control over the more fine-grained details of facial relative to body expressions. The total stimulus set comprises 4490 stimuli displaying seven different emotions (happy, angry, surprised, disgusted, fearful, sad, or neutral), depicted both from a frontal perspective (facing the observer) and from an averted view (averted to the left by 45°) to allow for a manipulation of the subjectively perceived face-to-face interaction. All stimuli have been validated and rated in terms of the naturalness of their appearance (for methodological details see Thoma et al., accepted). For the present study, in addition to neutral faces and bodies, stimuli from the categories “happy” and “angry” were included to represent positively and negatively valenced emotional categories. Only those frontal view pictures which, in the preceding validation studies, were recognized correctly by at least 80% of the pilot raters and received an average naturalness rating of at least 3 (on a scale ranging from *1* = *very unnatural* to *5* = *very natural*) were used in the present study to ensure that stimulus characteristics across categories were as comparable as possible. The corresponding averted view stimuli were selected to match the pre-selected frontal view pictures in terms of the depicted identities (see Figures 1, 2). All body and facial stimuli were embedded in 300 × 300 pixels JPEG files against a white background for the bodies and a black background for faces. In both experiments, all stimuli were shown against a white overall background.

### Procedure

Participants were seated in a light- and sound-attenuated room at a distance of 80 cm from a computer screen. The order of the experiments (bodies vs. faces) was counterbalanced across participants. The experimental design was based on a procedure described by Bediou et al. (2005), termed Faces Affect Identification Task. Two separate experiments were programmed for the body and face stimuli, each involving neutral, happy, and angry bodies/faces presented once from a frontal and once from an averted perspective. Each emotion category comprised 40 pictures (50% representing female faces/bodies), resulting in 240 trials per experiment (40 stimuli × 3 emotion categories × 2 perspectives). Presentation® software (Neurobehavioral Systems, Inc., Albany, CA, USA: http://neurobs.com) was used for stimulus presentation and the recording of the participants' responses.

**Figure 3:**
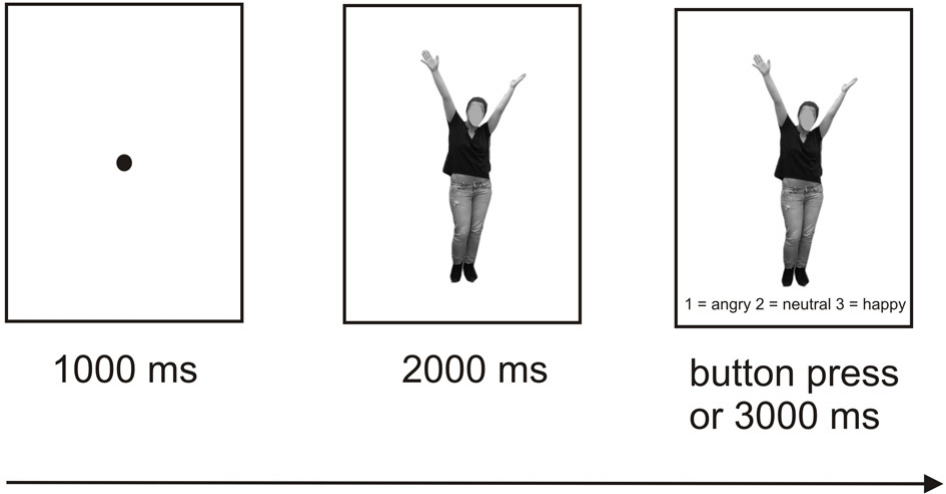
**Schematic illustration of the experimental design**.

Each trial started with the presentation of a fixation dot (1000 ms), followed by presentation of the stimulus alone (2000 ms) and subsequently of the stimulus along with the three response options presented below (1 = angry, 2 = neutral, 3 = happy) with a 3000 ms response window. All responses within this time window ended the current trial. The participants were instructed to determine by pressing the corresponding button which emotion was depicted by the respective body or face. In each of the two experiments, a short break was introduced after 120 trials lasting between 15 s and 10 min with the duration being controlled by the participant within these time limits. An illustration of the trial structure is provided in Figure 3. Each of the two experiments lasted between 20 and 25 min and was preceded by practice trials which could be repeated as often as necessary. The order of trial types involving the three different emotional valences and two perspectives was randomized. Only response times for correct trials entered the analyses.

### Questionnaire measures of empathy

Self-reported trait cognitive and affective empathy was assessed with the German translation (Schulte-Rüther et al., 2007) of the Balanced Emotional Empathy Scale (BEES: Mehrabian and Epstein, 1972) and the abbreviated German version (Paulus, 2007) of the Interpersonal Reactivity Index (IRI: Davis, 1980). The 30-item BEES measures the dispositional tendency to empathize with the emotional experiences of others, thus predominantly tapping affective empathy. In contrast to most other scales, it is balanced in terms of the positive and negative wording of items, thus controlling for social desirability effects. A total score was computed by subtracting the summed score for the negatively worded items from the positively worded items. The IRI comprises four subscales (Empathic Concern, Personal Distress, Perspective Taking, and Fantasy, comprising four items each in the German version) with the first two assessing affective and the last two assessing cognitive empathy components. The “Empathic Concern” subscale measures compassion and concern for unfortunate others, while “Personal Distress” assesses the tendency to experience discomfort in response to other people's suffering. The “Fantasy” scale measures the tendency to imaginatively transpose oneself into fictitious situations, e.g., when reading novels, while the “Perspective Taking” scale targets the ability to adopt other people's point of view in everyday life. Participants rated their agreement with each item on a bipolar 5-point rating scale (*“Does not describe me well” to “Describes me well”*). The summed scores per scale served as the dependent variables.

### Statistical analyses

#### Behavioral data

Response accuracy (number of correct responses) and response latency were submitted to separate repeated-measures analyses of variance (ANOVA) with Emotional Expression (happy, angry, neutral) and Perspective (frontal, averted) as factors.

#### EEG recordings and analysis

EEG was recorded from 30 Ag/AgCl electrodes mounted on an electrode cap according to the international 10/20 system (Jasper, 1958). Active electrodes were referenced to two linked mastoid electrodes and Fpz was used as a ground electrode. Electrode impedance was kept below 5 kΩ and EEG was digitalized at a sampling rate of 500 Hz. EEG signals were filtered with a bandpass filter of 0.5–35 Hz. Data were re-referenced to a common average reference. Independent component analysis (ICA) was used to control eye movements. Trials containing artifacts exceeding ± 50 μV were omitted from further analyses.

**Figure 4:**
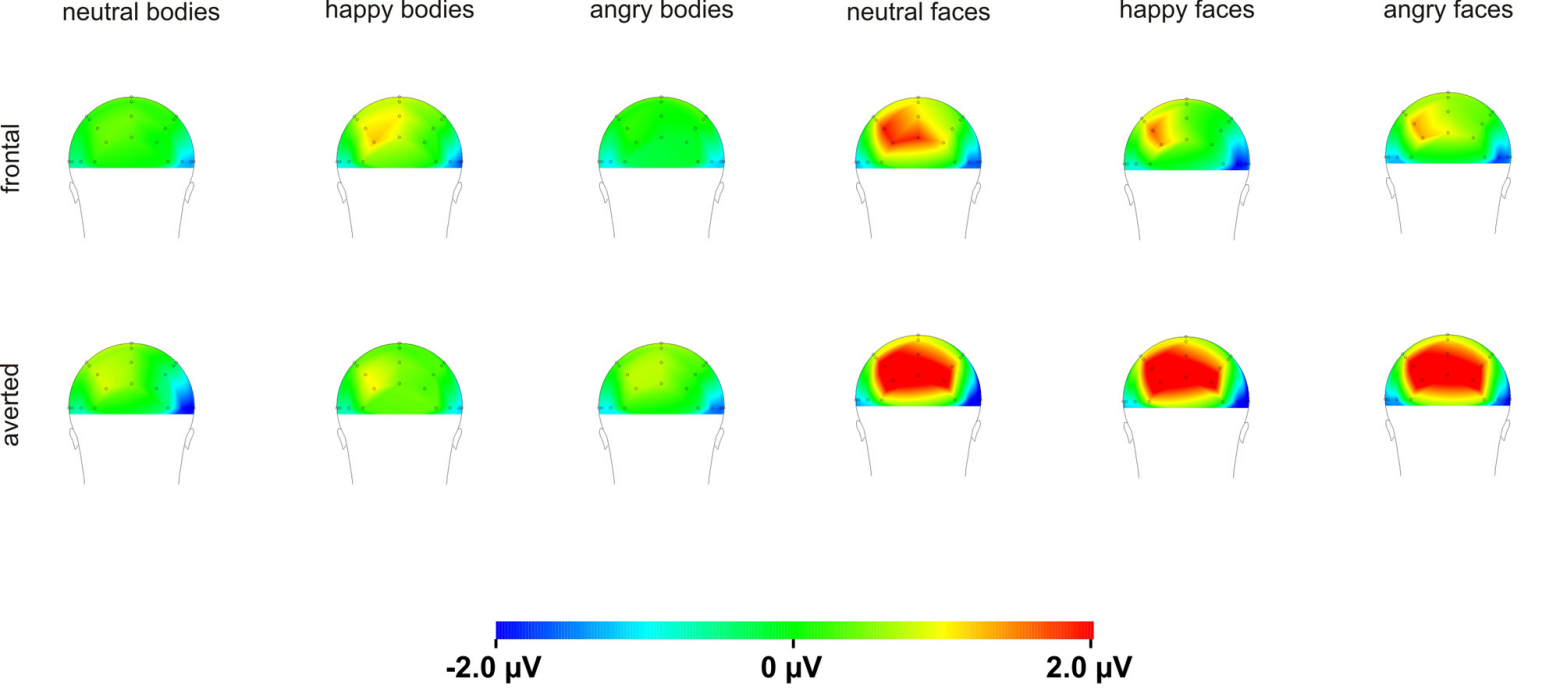
**Topography maps of the N170 for bodies and faces**.

Raw data were segmented offline in epochs lasting 450 ms starting 100 ms before and ending 350 ms after stimulus presentation. Data were baseline corrected by using the 100 ms before stimulus onset and averaged separately for each condition. Visual inspection suggested that the maximal or minimal amplitudes were seen at electrode positions P7/P8 and PO7/PO8 (see Figure 4), which is in line with a number of other studies linking the P100 (Thierry et al., 2006; Righart and de Gelder, 2007; Minnebusch et al., 2010) and the N170 (Bentin et al., 1999; Eimer, 2000a,b; Stekelenburg and de Gelder, 2004; Thierry et al., 2006; Righart and de Gelder, 2007) to these electrode positions. Maxima of P100 and N170 amplitudes were determined as the peak amplitude between 80 and 120 ms (P100) and 140–200 ms (N170) at electrode positions P7/P8 and PO7/PO8. Amplitude maxima were used to determine P100 and N170 latencies.

Amplitudes and latencies of the P100 and N170 were submitted to separate repeated-measure ANOVAs with Emotional Expression (happy, angry, neutral), Perspective (frontal, averted), Electrode Position (P7/P8, PO7/PO8), and Hemisphere (left, right) as factors. Greinhouse–Geisser corrections were used. ERPs were calculated for all trials, including those with incorrect responses, as the proportion of incorrect trials was overall rather low.

#### Correlations

The empathy scores (IRI Empathic Concern, Personal Distress, Perspective Taking, Fantasy, BEES total score) were correlated with the behavioral data (RTs and response accuracies for neutral, happy, and angry averted view and frontal view faces/bodies) and with the amplitudes and latencies of the P100 and N170 components for the respective stimulus groups.

## Results

### Behavioral data

Separate analyses were conducted for the accuracy scores and responses latencies (see Table 1 for descriptive data).

**Table 1:** **Response accuracies (% correct) and latencies (mean median ms) for the Body and Face Identification Tasks, computed separately for the two perspectives (frontal vs. averted) and three emotional expressions (neutral, happy, angry)**.

	**Bodies (*N* = 17)**		**Faces (*N* = 16)**	
	**% correct**	**RTs (ms)**	**% correct**	**RTs (ms)**
**FRONTAL**				
Neutral	97.8 (3.0)	337.8 (59.9)	92.3 (6.8)	379.3 (150.1)
Happy	90.3 (10.4)	362.6 (62.3)	89.2 (11.5)	396.3 (155.4)
Angry	79.9 (19.0)	402.9 (123.3)	89.7 (14.8)	394.3 (167.0)
**AVERTED**				
Neutral	94.4 (5.6)	362.0 (103.9)	92.2 (7.8)	388.9 (157.0)
Happy	87.2 (9.8)	383.0 (103.7)	85.0 (15.3)	423.6 (170.1)
Angry	79.6 (20.4)	420.3 (188.1)	90.8 (13.9)	419.8 (187.3)

#### Body identification task

Analysis of the accuracy scores in the Body Identification Task revealed main effects of Emotional Expression [*F*_(1.3, 22)_ = 11.0, p < 0.001] and Perspective [*F*_(1, 16)_ = 14.7, *p* = 0.001] with better performance for neutral compared to happy and angry body postures (both *p* ≤ 0.012, no significant difference for happy vs. angry emotions: *p* = 0.068) and for frontal compared to averted perspective body postures. There was no significant interaction between Emotional Expression and Perspective (*p* = 0.263). For response speed, none of the effects reached significance (all *p* ≥ 0.131).

#### Face identification task

For the accuracy scores, none of the comparisons reached significance (all *p* ≥ 0.115).

Analysis of response latencies yielded a main effect of Perspective [*F*_(1, 15)_ = 6.5, *p* = 0.022] with delayed response times for averted compared to frontal view faces, but no significant main effect of Emotional Expression and no significant interaction (both *p* ≥ 0.212).

### Electrophysiological data

**Figure 5:**
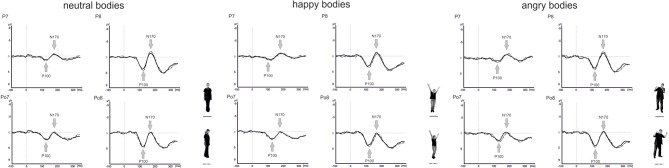
**Grand average wave forms for frontal and averted view neutral, happy, and angry bodies**.

**Figure 6:**
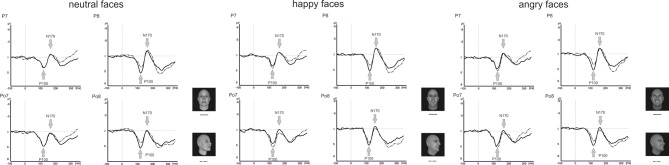
**Grand average wave forms for frontal and averted view neutral, happy, and angry faces**.

Grand-averaged ERPs for frontal and averted bodies and faces are depicted in Figures 5, 6. Separate analyses were conducted for the amplitudes and latencies of the P100 and N170.

#### Body identification task

For the P100 amplitude, there were main effects of Perspective [*F*_(1, 16)_ = 5.1, *p* = 0.039] and Hemisphere [*F*_(1, 16)_ = 12.8, *p* = 0.003] with enhanced P100 amplitudes for averted compared to frontal view bodies and in the right compared to the left hemisphere. There were no other significant main effects or interactions (all *p* ≥ 0.076).

Analysis of the P100 latencies showed main effects of Emotional Expression [*F*_(1.5, 24.6)_ = 5.6, *p* = 0.015] and Electrode Position [*F*_(1, 16)_ = 7.1, *p* = 0.017] with delayed P100 latencies for happy compared to neutral faces and at parieto-occipital electrodes compared to parietal electrode positions (see Figure 5). In addition, there was a significant Perspective × Electrode Position × Hemisphere interaction [*F*_(1, 16)_ = 7.9, *p* = 0.013]. To resolve the three-way interaction, separate analyses were performed for P100 latencies in the right and left hemisphere. In the right hemisphere, there was a main effect of Perspective [*F*_(1, 16)_ = 5.4, *p* = 0.034] with delayed latencies for averted view compared to frontal view bodies and a significant Perspective × Electrode Position interaction [*F*_(1, 16)_ = 5.3, *p* = 0.036]. Subsequent paired comparisons revealed delayed P100 latencies for averted compared to frontal view bodies at electrode position P7/P8 [*F*_(1, 16)_ = 8.7, *p* = 0.010]. None of the other comparisons reached significance (all *p* ≥ 0.108).

Analysis of the N170 amplitudes yielded a main effect of Electrode Position [*F*_(1, 16)_ = 15.3, *p* = 0.001] with enhanced N170 amplitudes at parietal electrodes compared to parieto-occipital electrode positions and significant interactions between Perspective and Hemisphere [*F*_(1, 16)_ = 14.2, *p* = 0.002] as well as Emotional Expression, Electrode Position, and Hemisphere [*F*_(2, 31.5)_ = 3.3, *p* = 0.049]. To resolve the three-way interaction, separate analyses were conducted at electrode positions P7/P8 and PO7/PO8. At electrode position P7/P8, there was a significant Emotional Expression × Hemisphere interaction [*F*_(1.9, 31)_ = 4.9, *p* = 0.015]. Subsequent comparisons revealed enhanced N170 amplitudes for angry compared to happy body postures in the left hemisphere [*F*_(1.9, 30.5)_ = 4.3, *p* = 0.022]. None of the other comparisons reached significance (all *p* ≥ 0.060).

None of the analyses involving the N170 latencies reached significance (all *p* ≥ 0.205).

#### Faces identification task

Analysis of the P100 amplitude yielded a Perspective × Hemisphere interaction [*F*_(1, 15)_ = 21.4, *p* < 0.001] with enhanced amplitudes for averted view compared to frontal view faces in the left hemisphere [*F*_(1, 15)_ = 12.3, *p* = 0.003]. The opposite pattern emerged in the right hemisphere with enhanced amplitudes for frontal view compared to averted view faces (see Figure 6).

None of the analyses involving P100 latencies reached significance (all *p* ≥ 0.113).

For the N170 amplitude, there was a main effect Electrode Position [*F*_(1, 15)_ = 23.0, *p* < 0.001] with enhanced N170 amplitudes at parietal electrodes compared to parieto-occipital electrodes. Furthermore, there were significant interactions between Perspective and Electrode Position [*F*_(1, 15)_ = 10.3, *p* < 0.001], Emotional Expression and Electrode Position [*F*_(1.9, 28.3)_ = 4.2, *p* = 0.024]. To resolve the three-way interaction, separate analyses were conducted for electrode positions P7/P8 and PO7/PO8. At electrode positions PO7/PO8, there was a main effect of Perspective with enhanced N170 amplitudes for frontal compared to averted view faces [*F*_(1, 15)_ = 4.7, *p* = 0.047]. At electrode positions P7/P8, none of the comparisons reached significance (all *p* ≥ 0.357).

Analysis of the N170 latencies revealed a main effect of Perspective [*F*_(1, 15)_ = 14.2, *p* = 0.002] with delayed N170 latencies for averted compared to frontal view faces. In addition, there were interactions between Emotional Expression and Electrode Position [*F*_(1.7, 26.2)_ = 5.5, *p* = 0.013], as well as Emotional Expression, Perspective, Electrode Position, and Hemisphere [*F*_(1.5, 22.4)_ = 3.9, *p* = 0.042]. To resolve the four-way interaction, separate analyses were performed at electrode positions P7/P8 and PO7/PO8. At both electrode positions, there was a main effect of Perspective with delayed N170 latencies for averted compared to frontal view faces [P7/P8: *F*_(1, 15)_ = 7.4, *p* = 0.016; PO7/PO8: *F*_(1, 15)_ = 8.2, *p* = 0.012]. None of the other comparisons reached significance (all *p* ≥ 0.210).

### Correlations

Behavioral data—accuracies and response latencies, computed separately for the two categories (faces vs. bodies), three emotions (neutral, happy, and angry), and perspectives (frontal vs. averted)—were correlated with the BEES total score and the IRI subscores (empathy, personal distress, perspective taking, and fantasy) (see Table 2 for the empathy scores in the two groups of participants performing the Body vs. Face Identification Tasks). Furthermore, the BEES and IRI scores were correlated with P100 and N170 amplitudes and latencies, summed up across electrode positions (P7 + P8 + P07 + PO8), separately for the two categories, three emotions, and two perspectives. All correlations were computed using Pearson correlations.

**Table 2:** **Mean scores (standard deviations) on the Balanced Emotional Empathy Scale (BEES; Mehrabian and Epstein, 1972) and on the abbreviated German version of the Interpersonal Reactivity Index (IRI, Paulus, 2007), calculated separately for the participants in the body and in the face experiment**.

	**Body experiment (*N* = 17)**	**Faces experiment (*N* = 16)**
**BEES**		
Total Score	48.12 (27.13)	53.50 (25.47)
**IRI**		
Perspective taking	15.59 (3.04)	16.00 (3.03)
Fantasy	13.18 (3.28)	13.31 (3.80)
Empathic concern	14.47 (2.55)	14.37 (2.45)
Personal distress	10.59 (3.40)	9.44 (2.58)

#### Correlations on a behavioral level

On the Body Identification Task, significant associations emerged between the BEES total score and the percentage of correct responses for the frontal view (*r* = 0.650, *p* = 0.005) and averted view (*r* = 0.554, *p* = 0.021) angry body expressions. IRI personal distress correlated significantly with accuracy scores for neutral frontal (*r* = −0.581, *p* = 0.014) and angry averted (*r* = −0.533, *p* = 0.033) bodies. On the Face Identification Task, BEES total scores correlated significantly with response latencies for all stimulus categories (neutral frontal: *r* = −0.580, *p* = 0.018/neutral averted: *r* = −0.585, *p* = 0.017/happy frontal: *r* = −0.638, *p* = 0.008/happy averted: *r* = −0.586, *p* = 0.017/angry frontal: *r* = −0.628, *p* = 0.009/angry averted: *r* = −0.596, *p* = 0.015). IRI perspective taking scores correlated significantly with accuracy scores for frontal happy faces (*r* = 0.524, *p* = 0.037). There were no other significant correlations (all *p* ≥ 0.053).

#### Correlations with P100 and N170 amplitudes

IRI personal distress scores correlated significantly with the amplitude of the N170 for the frontal view angry (*r* = 0.532, *p* = 0.028) and for the averted view happy (*r* = 0.495, *p* = 0.044) bodies. IRI empathic concern subscores correlated significantly with the N170 amplitude for the averted neutral (*r* = −0.546, *p* = 0.015) and for angry faces, presented both in frontal (*r* = −0.549, *p* = 0.034) and in averted view (*r* = −0.566, *p* = 0.028). There were no other significant associations between BEES/IRI scores and P100 or N170 amplitudes on the Body and Faces Identification Tasks (all *p* ≥ 0.056).

#### Correlations with P100 and N170 latencies

IRI perspective taking scores correlated significantly with P100 latencies for averted happy (*r* = −0.593, *p* = 0.020) and averted angry faces (*r* = −0.552, *p* = 0.033). P100 latencies for neutral frontal faces correlated significantly with IRI empathic concern (*r* = −0.706, *p* = 0.003) and IRI personal distress (*r* = −0.570, *p* = 0.027), and IRI fantasy scores correlated significantly with N170 latencies for averted neutral faces (*r* = −0.627, *p* = 0.009). P100 latencies for angry averted bodies correlated significantly with IRI perspective taking scores (*r* = −0.542, *p* = 0.030). There were no other significant correlations (all *p* ≥ 0.051).

**Figure 7:**
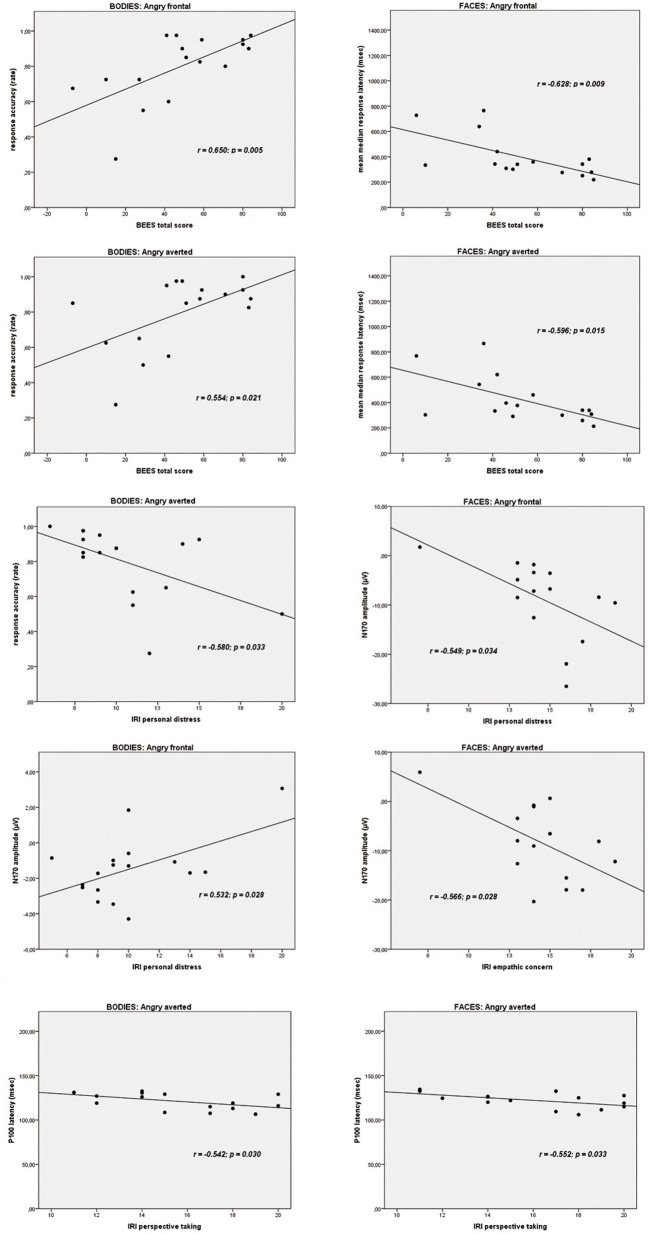
**Scatterplots depicting the correlations between trait empathy scores (BEES, Balanced Emotional Empathy Scale; IRI, Interpersonal Reactivity Index) and behavioral data and ERPs to angry emotional expressions (bodies and faces)**.

As angry emotional expressions were most consistently involved in the correlations reported above, and as these are of particular theoretical interest to us, scatterplots for these correlations are highlighted in Figure 7.

## Discussion

The aim of the present study was to elucidate differences in the processing of bodies and faces showing neutral, happy, and angry expressions, presented from an averted relative to a frontal view perspective, in relation to trait empathy.

Regarding the effect of the valence of distinct emotions expressed by bodies and faces, response accuracy was higher for neutral relative to happy and angry bodies. Although, we aimed for creating non-static neutral body expressions (e.g., by asking the non-professional actors to perform everyday actions such as combing their hair when posing for the neutral expressions), it might still be the case that these expressions were overall characterized by less movement than the emotional expressions. This might have rendered it easier to differentiate the neutral from the emotional bodies correctly. On an electrophysiological level, P100 latencies were delayed for happy relative to neutral body expressions and N170 amplitudes were enhanced for angry relative to happy body expressions, but only for left-hemispheric electrode positions.

Overall, the fact that processing differences between different emotional valences were not very strongly pronounced in our study might partly be linked to the fact that within each category (bodies and faces) stimuli were very well matched across the three different valences (neutral, angry, and happy) in terms of the identification accuracy and perceived naturalness, as determined in a prior pilot study (Thoma et al., accepted). Also, in contrast to other EEG studies (Hadjikhani and de Gelder, 2003; Stekelenburg and de Gelder, 2004; Meeren et al., 2005; van Heijnsbergen et al., 2007), we not only included negative emotions or compared negative with neutral emotions, but also included a positive (happy) emotion category.

As far as distinct processing of frontal view relative to averted view bodies and faces is concerned, performance was more accurate for frontal relative to averted view bodies, while responses were faster for frontal relative to averted faces. This is in line with the expected performance drop for averted relative to frontal view presentations of stimuli, although it is interesting that this manifests itself differently for the two stimulus categories, primarily affecting response accuracy for bodies and performance speed for faces. However, both response patterns indicate that emotional expressions of frontal view bodies and faces are easier to recognize compared to emotional expressions of averted view presentation. This is in line with suggestions that frontal view presentations of emotional bodies and faces might induce a feeling of being involved in a direct interpersonal interaction in the observer. This might help participants to infer the intended emotional action (bodies) or the emotional mental state (faces) (see de Gelder et al., 2010) of the interaction partner by triggering empathic resonance mechanisms more easily. The ecological relevance of the distinction between faces with frontal vs. averted gaze relative to the observer has been e.g., illustrated by Kampe et al. (2001). They demonstrated that when eye gaze was directed at the participants, activity in the ventral striatum as part of the brain reward system, correlated positively with the perceived attractiveness of the face. On the other hand, this correlation was negative, when eye gaze was averted. Also, activity associated with direct vs. averted gaze was observed in the right paracingulate cortex and the left temporal pole, two areas associated with mentalizing abilities (Kampe et al., 2003).

The current results stand in contrast to those in the study of Schulte-Rüther et al. (2007). They reported similar response times for frontal compared to averted view stimulus presentation. A possible explanation for this effect might be that Schulte-Rüther et al. (2007) focused on the response times, while accuracy scores were not analyzed. It is possible that subjects showed the same response times for frontal and averted view faces but performed better for frontal view faces. Thus, the behavioral data of Schulte-Rüther et al. (2007) did not clarify satisfactorily whether there are processing differences between frontal and averted view faces.

In our study, P100 amplitudes were enhanced over parieto-occipital and parietal brain areas for averted relative to frontal bodies (see Figure 4), and P100 latencies were delayed for averted relative to frontal bodies for parietal electrode positions and for right-hemispheric electrodes. As the P100 has been linked to early stimulus categorization processes (Herrmann et al., 2005, for a review see Minnebusch and Daum, 2009), these effects might be associated with the increased difficulty of classifying stimuli as bodies/faces when they are not presented facing the observer and thus not all stimulus features can be fully perceived. According to this, it is possible that the detection of a stimulus as a body might take less time if the stimulus is presented from a frontal compared to an averted view. The P100 amplitude might be enhanced as the activation of additional areas is necessary for the successful processing of averted view bodies. In the fMRI study by Schulte-Rüther et al. (2007), there were no activation differences linked to direct vs. averted view stimuli beyond early visual processing areas, which might be partly due to the poor time resolution inherent to fMRI technology. The results by Schulte-Rüther et al. (2007) are in line with our findings of a modulation of early components, such as the P100, but not of later components, like the N170 for bodies highlighting the advantage of a more fine-grained temporal analysis. However, Schulte-Rüther et al. (2007) aimed to explore which brain regions are related to emotional and view-dependent faces processing, whereas the current investigation elucidated the time course of emotional and view-dependent face and body processing, emphasizing the importance of combining these methods. The N170 might not be modulated by the perspective of the presented stimuli as this component is linked to configural processing mechanisms. Configural processing relies on the relative spatial distance between stimulus features and is important for the identification of other people. For faces, the result pattern was more complex in that averted relative to frontal view faces elicited enhanced P100 amplitudes for left-hemispheric electrode positions, while it was the other way round for right-hemispheric electrode positions. Thus, in the time window of the P100, we found the same activation patterns for faces and bodies in the left hemisphere but opposite patterns in the right hemisphere. N170 amplitudes were enhanced for frontal relative to averted faces at parieto-occipital, but not at parietal electrode positions and N170 latencies were delayed for averted relative to frontal faces. To our knowledge, there are no previous EEG-studies examining the effects of bodies being presented from a frontal vs. averted perspective on the P100/N170. Previous face processing studies focused on direct vs. averted gaze rather than on direct vs. averted head orientations like we did. Overall, findings are rather inconsistent in terms of whether these components are modulated by direct vs. averted gaze and there is even less consistency in terms of whether effects are lateralized over the right or left hemisphere (see review by Itier and Batty, 2009).

Our third aim was to elucidate the relationship between trait empathy, in terms of a personality-related “chronic” trait, and the processing of bodies and faces. The correlations of most theoretical interest to us, most consistently involving angry expressions, are presented in Figure 7. The pattern of correlations on a behavioral level is in line with the suggestion of de Gelder et al. (2010) that facial expressions might be more strongly related to an individual's mental state, as higher affective trait empathy correlated significantly with faster responses for all facial categories (neutral, happy, angry, presented both from a frontal, and from an averted perspective), while for body stimuli, correlations between affective trait empathy and higher performance accuracy emerged most consistently for angry bodies. This is again plausible in light of de Gelder et al. (2010) suggestion that emotional body expressions might be more strongly linked to emotional action preparation. An angry body expression observed in an interaction partner might trigger a strong empathic response in the observer, and the ability to affectively share and respond to this emotion might be particularly relevant in that an appropriate action (e.g., flight when being attacked by an angry opponent) might have to be prepared. Accordingly, more pronounced affective trait empathy also correlated most consistently with larger N170 amplitudes for angry emotional expressions, in particular for frontal view bodies and for both averted and frontal view faces. However, it is noteworthy that the two affective empathy components empathic concern and personal distress were differentially related to N170 components for bodies and faces. While empathic concern was negatively related to the N170 amplitudes to angry facial expressions in particular, the correlation between IRI personal distress and frontal angry and averted happy expressions was positive. It is plausible that a higher disposition to feel personal distress in response to negative emotions such as anger, might be related to increased N170 amplitudes to frontally presented emotional body expressions of anger but not in association with angry facial expressions, because—again interpreted in light of de Gelder et al.'s model (2010)—action preparation (e.g., flight) appears more relevant in the first context. On the other hand, within the same theoretical framework, it also appears to make sense that a more pronounced tendency to feel for another person's mental state, i.e., empathic concern, might be associated with a downregulation of neural responses to angry faces with which one usually does not empathize. It is more difficult to explain the positive association between IRI personal distress and N170 amplitudes to averted happy faces and the negative correlation between IRI empathic concern and N170 amplitudes to averted neutral faces in a similar way. However, it is possible that, presented from an averted perspective, these expressions might have more easily been confused with negative emotional expressions. For instance, it has been shown in previous studies that neutral and sad expressions are often confused (Du and Martinez, 2011).

A further rather consistent pattern emerged for the significant correlations between cognitive trait empathy and shorter P100 latencies for averted happy faces, averted angry faces, and bodies and averted neutral faces. It is plausible that better cognitive empathic abilities, i.e., the ability to cognitively infer someone else's mental state, might aid in the faster extraction of the emotional features from a stimulus, as reflected by the P100 (Meeren et al., 2005; Pourtois et al., 2005; Righart and de Gelder, 2007; van Heijnsbergen et al., 2007), particularly when such a stimulus is presented from an averted perspective.

One potential shortcoming of our study might consist in the fact that we only assessed “chronic” trait empathy but not acute empathic reactivity to the happy vs. angry faces shown. In future studies, it would be desirable to address this further. In this context, it would also be interesting to see whether the empathic response to emotional body expressions with blurred faces is comparable to the one shown in response to emotional virtual facial avatars.

Also, it would have been desirable to analyze behavioral data and ERPs for bodies vs. faces in one ANOVA to highlight the effects of perspective and emotional valence, but this was not statistically possible in our study, as only 11 participants performed both the body and the faces task. In future studies, it would be desirable to include body and face stimuli in the same experiment, preferably either both representing real-life photographs or virtual avatars to increase comparability.

Taken together, our results highlight the role of trait empathy in the perception of emotional faces and bodies, although further studies are necessary to elucidate and replicate this pattern of results.

### Conflict of interest statement

The authors declare that the research was conducted in the absence of any commercial or financial relationships that could be construed as a potential conflict of interest.

## References

[B1] Batty M. Taylor M. J. (2003). Early processing of the six basic facial emotional expressions. Brain Res. Cogn. Brain Res. 17, 613–620 10.1016/S0926-6410(03)00174-5 14561449

[B2] Bediou B. Franck N. Saoud M. Baudouin J. Y. Tiberghien G. Dalery J. (2005). Effects of emotion and identity on facial affect processing in schizophrenia. Psychiatry Res. 133, 149–157 10.1016/j.psychres.2004.08.008 15740991

[B3] Bentin S. Deouell L. Y. Soroker N. (1999). Selective visual streaming in face recognition: evidence from developmental prosopagnosia. Neuroreport 10, 823–827 10.1016/j.neuropsychologia.2005.03.017 10208555

[B4] Davis M. (1980). A multidimensional approach to individual differences in empathy. JSAS Catalog. Sel. Doc. Psychol. 10, 85

[B5] Decety J. Jackson P. (2004). The functional architecture of human empathy. Neurosci. Biobehav. Rev. 3, 71–100 10.1177/1534582304267187 15537986

[B6] de Gelder B. (2006). Towards the neurobiology of emotional body language. Nat. Rev. Neurosci. 7, 242–249 10.1038/nrn1872 16495945

[B7] de Gelder B. Van den Stock J. Meeren H. K. Sinke C. B. Kret M. E. Tamietto M. (2010). Standing up for the body. Recent progress in uncovering the networks involved in the perception of bodies and bodily expressions. *Neurosci. Biobehav. Rev.* 34, 513–527 10.1016/j.neubiorev.2009.10.008 19857515

[B8] Du S. Martinez A. M. (2011). The resolution of facial expressions of emotions. J. Vis. 11, 24 10.1167/11.13.24 22131445 PMC3702732

[B9] Eimer M. (2000a). Effects of face inversion on the structural encoding and recognition of faces. Evidence from event-related brain potentials. Brain Res. Cogn. Brain Res. 10, 145–158 10.1016/S0926-6410(00)00038-0 10978702

[B10] Eimer M. (2000b). The face-specific N170 component reflects late stages in the structural encoding of faces. Neuroreport 11, 2319–2324 10923693 10.1097/00001756-200007140-00050

[B11] Gallese V. Keysers C. Rizzolatti G. (2004). A unifying view of the basis of social cognition. Trends Cogn. Sci. 8, 396–403 10.1016/j.tics.2004.07.002 15350240

[B12] Grezes J. Pichon S. de Gelder B. (2007). Perceiving fear in dynamic body expressions. Neuroimage 35, 959–967 10.1016/j.neuroimage.2006.11.030 17270466

[B13] Hadjikhani N. de Gelder B. (2003). Seeing fearful body expressions activates the fusiform cortex and amygdala. Curr. Biol. 13, 2201–2205 14680638 10.1016/j.cub.2003.11.049

[B14] Herrmann M. J. Ehlis A. C. Muehlberger A. Fallgatter A. J. (2005). Source localization of early stages of face processing. Brain Topogr. 18, 77–85 10.1007/s10548-005-0277-7 16341576

[B15] Itier R. J. Batty M. (2009). Neural bases of eye and gaze processing: the core of social cognition. Neurosci. Biobehav. Rev. 33, 843–863 10.1016/j.neubiorev.2009.02.004 19428496 PMC3925117

[B16] Jasper H. H. (1958). Report of the committee on methods of clinical examination in electroencephalography. Electroencephalogr. Clin. Neurophysiol. 10, 370; reprinted: (1961) in Am. J. Technol. 1–13.

[B17] Kampe K. K. Frith C. D. Dolan R. J. Frith U. (2001). Reward value of attractiveness and gaze. Nature 413, 589 10.1038/35098149 11595937

[B18] Kampe K. K. Frith C. D. Frith U. (2003). “Hey John”: signals conveying communicative intention toward the self activate brain regions associated with “mentalizing,” regardless of modality. J. Neurosci. 23, 5258–5263 12832550 10.1523/JNEUROSCI.23-12-05258.2003 PMC6741156

[B19] Kret M. E. Pichon S. Grezes J. de Gelder B. (2011). Similarities and differences in perceiving threat from dynamic faces and bodies. An fMRI study. Neuroimage 54, 1755–1762 10.1016/j.neuroimage.2010.08.012 20723605

[B20] Meeren H. K. van Heijnsbergen C. C. de Gelder B. (2005). Rapid perceptual integration of facial expression and emotional body language. Proc. Natl. Acad. Sci. U.S.A. 102, 16518–16523 10.1073/pnas.0507650102 16260734 PMC1283446

[B21] Mehrabian A. Epstein N. (1972). A measure of emotional empathy. J. Pers. 40, 525–543 4642390 10.1111/j.1467-6494.1972.tb00078.x

[B22] Minnebusch D. Keune P. Suchan B. Daum I. (2010). Gradual inversion affects processing of human body shapes. Neuroimage 49, 2746–2755 10.1016/j.neuroimage.2009.10.046 19853043

[B23] Minnebusch D. A. Daum I. (2009). Neuropsychological mechanisms of visual face and body perception. Neurosci. Biobehav. Rev. 33, 1133–1144 10.1016/j.neubiorev.2009.05.008 19500617

[B24] Morris J. S. Frith C. D. Perrett D. I. Rowland D. Young A. W. Calder A. J. (1996). A differential neural response in the human amygdala to fearful and happy facial expressions. Nature 383, 812–815 10.1038/383812a0 8893004

[B25] Paulus C. (2007). Saarbrücker Persönlichkeitsfragebogen. Saarbrücken: Universität des Saarlandes

[B25a] Pourtois G. Thut G. Grave de Peralta R. Michel C. Vuilleumier P. (2005). Two electrophysiological stages of spatial orienting towards fearful faces: early temporo-parietal activation preceding gain control in extrastriate visual cortex. Neuroimage 26, 149–163 10.1016/j.neuroimage.2005.01.015 15862215

[B26] Righart R. de Gelder B. (2007). Impaired face and body perception in developmental prosopagnosia. Proc. Natl. Acad. Sci. U.S.A. 104, 17234–17238 10.1073/pnas.0707753104 17942679 PMC2040466

[B27] Rossion B. Gauthier I. Tarr M. J. Despland P. Bruyer R. Linotte S. (2000). The N170 occipito-temporal component is delayed and enhanced to inverted faces but not to inverted objects: an electrophysiological account of face-specific processes in the human brain. Neuroreport 11, 69–74 10683832 10.1097/00001756-200001170-00014

[B28] Rotshtein P. Malach R. Hadar U. Graif M. Hendler T. (2001). Feeling or features: different sensitivity to emotion in high-order visual cortex and amygdala. Neuron 32, 747–757 11719213 10.1016/s0896-6273(01)00513-x

[B29] Schulte-Rüther M. Markowitsch H. J. Fink G. R. Piefke M. (2007). Mirror neuron and theory of mind mechanisms involved in face-to-face interactions: a functional magnetic resonance imaging approach to empathy. J. Cogn. Neurosci. 19, 1354–1372 10.1162/jocn.2007.19.8.1354 17651008

[B30] Shamay-Tsoory S. G. (2011). The neural bases for empathy. Neuroscientist 17, 18–24 10.1177/1073858410379268 21071616

[B31] Singer T. Lamm C. (2009). The social neuroscience of empathy. Ann. N.Y. Acad. Sci. 1156, 81–96 10.1111/j.1749-6632.2009.04418.x 19338504

[B32] Stekelenburg J. J. de Gelder B. (2004). The neural correlates of perceiving human bodies: an ERP study on the body-inversion effect. Neuroreport 15, 777–780 15073513 10.1097/00001756-200404090-00007

[B33] Thierry G. Pegna A. J. Dodds C. Roberts M. Basan S. Downing P. (2006). An event-related potential component sensitive to images of the human body. Neuroimage 32, 871–879 10.1016/j.neuroimage.2006.03.060 16750639

[B35] van de Riet W. A. Grezes J. de Gelder B. (2009). Specific and common brain regions involved in the perception of faces and bodies and the representation of their emotional expressions. Soc. Neurosci. 4, 101–120 10.1080/17470910701865367 19255912

[B36] van Heijnsbergen C. C. Meeren H. K. Grezes J. de Gelder B. (2007). Rapid detection of fear in body expressions, an ERP study. Brain Res. 1186, 233–241 10.1016/j.brainres.2007.09.093 17996856

[B37] Varga A. C. (1975). Declaration of Helsinki (Adopted by the 18th world medical assembly in Helsinki, Finland, and revised by the 29th world medical assembly in Tokyo, 1975), in The Main Issue in Bioethics, Revised edition, (New York, NY: Paulist Press, 1984).

[B38] Vuilleumier P. Pourtois G. (2007). Distributed and interactive brain mechanisms during emotion face perception: evidence from functional neuroimaging. Neuropsychologia 45, 174–194 10.1016/j.neuropsychologia.2006.06.003 16854439

